# The impact of video game playing on Chinese adolescents’ academic achievement: Evidence from a moderated multi-mediation model

**DOI:** 10.1371/journal.pone.0313405

**Published:** 2024-11-19

**Authors:** Xiaoxia Gu, Norlizah Che Hassan, Tajularipin Sulaiman, Zhixia Wei, Jingyi Dong

**Affiliations:** 1 Department of Foundations of Education, Faculty of Educational Studies, Universiti Putra Malaysia, Serdang, Selangor, Malaysia; 2 Hebei Key Laboratory of Children’s Cognition and Digital Education, School of Educational Studies, Langfang Normal University, Langfang, Hebei, China; University of Connecticut Health Center: UConn Health, UNITED STATES OF AMERICA

## Abstract

Internet access for adolescents is becoming more prevalent around the world. Although video game playing has been verified to be negatively related to adolescent academic achievement, the mechanisms underlying this relationship are also unknown. Using a nationally representative sample of adolescents from the China Education Panel Survey (2014–2015), this study aims to explore the parallel mediation roles of self-educational expectation and learning attitude in the link between video game playing and academic achievement, and whether the direct and indirect effects are moderated by parent-child relationship. The results indicate that video game playing in adolescents is both directly and indirectly related to their academic achievement, and self-educational expectation and learning attitude partially mediate this association. Moreover, the results reveal that parent-child relationship moderates the direct association between video game playing and academic achievement as well as the indirect association of video game playing on academic achievement via self-educational expectation, respectively. By showing empirical evidence for the usefulness of social cognitive theory to adolescents’ academics in the Internet Age, our research provides a supplement to existing literature.

## 1. Introduction

The rapid development of internet technology has brought about changes worldwide, and computers have become an important part of the daily life of adolescents. They are in a hyper-connected world where constant digital multitasking results in a unique social environment [1]. Thus, video gaming has become a widespread and popular activity among young people across various countries [2–6]. In recent years, the online game industry has been developing particularly rapidly in China, and the number of users continues to climb, with a marked increase in adolescents. As of June 2023, the total number of internet users in China amounted to 1.079 billion, with an internet penetration rate of 76.4%. Within this group, the number of online game users reached 550 million, accounting for 51.0%. Meanwhile, teenagers aged 10 to 19 accounted for 13.9% of the total Netizen population [7]. In particular, it should be noted that minors are the most loyal users of online games. In China, the number of underage internet users was 193 million in 2022, and the internet penetration rate reached 97.2%. Among them, the proportion of internet users who regularly play games online reached 67.8%, up 5.5 percentage points from 2021 (62.3%), showing an upward trend [8]. In a word, online gaming has seamlessly integrated into our daily lives and internet use and video game playing have become common activities for adolescents.

Furthermore, the influence of video games on youth development, particularly in academics and behaviors, has attracted considerable attention around the world. One of the risk factors is that some adolescents devote a lot of time and energy to online games leading to internet addiction. There is a rich body of evidence suggesting that adolescent gaming addiction is relatively pronounced, with rates reported to be around 8% in U.S. [9], 10% in Hong Kong [10], 15% in Italy [11], and 16% in Saudi Arabia [12]. In particular, some longitudinal studies indicated that once adolescents become addicted to internet use and video game playing, the symptoms tend to persist over time [13, 14]. Stevens et al. pointed out that gaming disorder has emerged as a significant global prevalence, affecting adolescents and young adults across various cultures and societies [15]. Other studies have verified the harmful consequences of video game addiction, such as endangering physical and mental health [16–18], increasing aggressive behavior [19, 20], affecting academic performance [21, 22]. Hence, people are becoming more aware of the problems of excessive video games.

Regarding the impact of video games on adolescents’ academic achievement, some studies have confirmed their positive effects, such as enhancing cognitive skills and relieving stress [23, 24], but the drawbacks are more obvious, as frequent video games tend to be negatively correlated with academic performance [21, 22, 25–28]. Adolescence is considered a critical and tumultuous period during which teenagers are establishing their identities and personalities and searching for meaning in their lives. As the famous psychologist Erikson stated, adolescents are exposed to the conflict between self-identity and role confusion [29]. The internet and video games are prone to have a distorting and alienating influence on adolescents whose personalities have not yet matured. Other studies also identified that students are considered to be the group most susceptible to encountering issues with internet usage [30, 31]. Adolescents, navigating unclear role identities and an evolving self-concept, may increasingly turn to internet activities for engagement, which can be accompanied by a declining interest in school life. When facing difficulties with self-control, they tend to overuse of the internet and social media and become addicted to the internet [32, 33]. These are important aspects that reduce students’ concentration and affect their academic success. Therefore, the negative impact of the internet and video games on adolescents’ academic achievement cannot be ignored.

Individuals, as the principal part, play an important role in the effect of video game playing on academic achievement among adolescents. A few studies have explored the pathways in which video games affect academic achievement, such as academic engagement [34, 35], and self-efficacy [36]. Drummond et al. pointed out that playing video games itself does not appear to affect academic performance, and research is more often needed to clarify the hypothesized theoretical frameworks that support potential mechanisms [37]. By taking a more detailed approach to understanding the effects of specific types of media and different engagement on more stratified groups, it is easier to ensure possible ways to influence learning. However, there is limited research on the intrinsic factors that shape the relationship between video games and academic achievement, particularly from the perspective of combining individuals and families. Thus, the focus of this study is how video game playing relates to adolescents’ academic achievement. What are the roles of self-educational expectation and learning attitude at the individual level and parent-child relationship at the family level in this influence pathway? To address this research gap, this study focuses on a group of Chinese junior high school students who play video games, examines the relationship between adolescents’ video game playing, educational expectation, learning attitude, and academic achievement, and explores the role that parent-child relationship plays in these mechanisms.

## 2. Literature review and hypotheses development

### 2.1 Game playing and academic achievement

There have been two opposing views on the value judgment of video games. One side affirms video games, insisting that they have positive effects on people’s lives, studies, and work. For example, Gee argued that video games allow individuals to discover social roles, understand social rules, recreate themselves, explore deeper learning styles, and improve comprehension and cognitive abilities [38, 39]. However, the other side takes the opposite stance, believing that video games are driven by commercial interests and adversely affect people’s lives and work, such as making young people addicted to games, neglecting their studies, alienating interpersonal relationships, confusing values and even committing crimes. According to Herz, video games eat up our time, win our hearts, and change our minds, leaving the players in a state of “control” [40]. In other words, while video games give us pleasure, they also make us more controlled by the game, even for adolescents.

The findings on the association between video game playing and academic achievement are also divided, with the relationship depending on different ways in which adolescents access the internet. On the one hand, some evidence suggests that good puzzle video games help promote students’ thinking skills and facilitates academic development [38, 39, 41, 42]. According to Willoughby, moderate internet use was related to a more positive academic orientation compared with both non use and high levels of use [43]. Nevertheless, on the other hand, the negative link between these two is well-recognized. Studies have shown that commercial electronic casual games cause unfavourable effects on adolescents’ academic achievement [25, 44, 45]. Particularly, Adelantado-Renau et al. revealed that the negative effects of screen activities such as watching television and playing video games on adolescents appear to be more significant than those on children [26]. Moreover, other studies proved that longer video game time [27], and more severe problematic internet use, such as gaming addiction [21, 22, 46], were significantly associated with lower academic achievement. Thereby, frequent video game playing among adolescents is a risk factor for unfavorable academic achievement. Therefore, we proposed the following hypothesis:

Hypothesis 1: Video game playing is negatively related to adolescents’ academic achievement.

### 2.2 Parallel mediation model

An increasing number of psychological theories are being used to explain human behavior, in which the role of self-processes is continually being emphasized since most external influences work through intermediary self-processing rather than directly affecting human functioning [47]. Currently, the social cognitive theory (SCT), developed by Bandura, stands as one of the most widely used theories for understanding changes in human behavior. Specifically, SCT argues that human behavior is shaped by the interplay of personal factors (e.g., cognitions, beliefs, and self-efficacy), environmental influences (e.g., others’ behaviors and feedback), and behavioral change (e.g., past behavior, efforts, and persistence) [48]. This theory reveals that individual behavior is influenced not only by external environmental factors but also regulated by internal cognitive processes. Based on this, SCT proposes that human behavior is purposefully goal-driven and regulated through a balance of control over internal cognitions and external influences [49].

As with other human behaviors, video game behavior is affected by a complex interaction of cognitive, environmental, and behavioral factors. It can be believed that Bandura’s social cognitive theory provides a fruitful and broad framework for understanding adolescents’ psychological and behavioral engagement in video games. Van Rooij et al. conducted a comprehensive review of existing popular theories based on children’s multidimensional motives for starting, continuing, and stopping video game playing, noting that social cognitive theory is one of the theoretical approaches [50]. Prior research has illustrated that individual-level cognitive structures (e.g., expectancy and self-regulation) are widely recognized as being closely related to internet and gaming behaviors in studies of adolescent development in the digital age [51, 52]. For example, studies found that problematic video gaming and internet use has significant effects on self-esteem [53, 54], self-efficacy [55], and self-concept [56]. Furthermore, other evidence has confirmed that video gaming can influence a range of outcomes by acting on internal personal factors, such as well-being [57, 58], procrastination [59], and academic achievement [36].

A recent study suggested that the concept of self is becoming increasingly important for understanding the psychological mechanisms underlying problematic online games [60]. Buckley and Anderson believed that individuals can acquire a range of complex behaviors, attitudes, expectations, beliefs, and perceptual schemas through their observation and participation in video games [61]. This experience in virtual world probably has a significant impact on adolescents’ cognitive development. Following the above mentioned social cognitive theory, personality systems typically cover elements such as values, expectations, beliefs, attitudes, and orientations of one’s own and others. These dispositions are often viewed as factors that regulate an individual’s behavior rather than as descriptions of habitual behavior [47]. What’s more, researchers have noted that the major factors influencing an individual’s behavior within a given domain are self-efficacy, outcome expectations, utility beliefs, and interests in the same and related domains [62, 63]. In this sense, self-educational expectation and learning attitude are important components of social cognition, and they can be reflected as a degree of engagement in learning. Given that both self-educational expectation and learning attitude are key predictors of adolescents’ academic success, this study examines the simultaneous mediating roles of these two variables within the framework of social cognitive theory. This approach not only provides a more comprehensive picture of the factors that may influence adolescents’ academic achievement but also allows for a deeper exploration of the internal mechanisms that shape the relationship between video game playing and academic achievement.

According to social cognitive theory, self-educational expectation, which refers to the realistic goals set by people based on their cognition and understanding of social functioning, is considered to be the most effective predictor of educational attainment [64, 65]. Prior studies have indicated that individuals’ educational aspirations in the early academic years have a significant impact on both their future academic achievement and educational attainment, and it serves as a form of psychological energy that can drive young people to actively pursue academic success [66, 67]. The difference between the highly exciting gaming sessions and the real world with heavy academic pressure can easily cause a psychological gap in adolescents, and excessive internet use may bring about risks such as academic burnout and even school dropout [68, 69]. Furthermore, online games lead players to follow specific paths and to be “controlled” [40], which can easily erode their aspirational beliefs. In such circumstances, the most easily lost beliefs among middle school students are giving up diligent studying and lowering self-expectations.

In addition, attitudes are usually interpreted as positive or negative emotions and thoughts associated with specific social objects, which are the result of emotions, thoughts, and behavioral tendencies due to previous experiences [70]. Attitudes, as a state of mental readiness and relatively enduring organization, are both a prerequisite and a consequence of behavior. Reacting positively to a situation with a positive attitude has a different impact on events and phenomena, as does reacting negatively to a situation with a negative attitude [71]. Previous empirical studies have shown that the extent of effort demonstrated by students in achieving learning goals, as a positive or negative learning attitude, is considered a predictive factor for their academic success [72–74]. In this context, we argue that adolescents, with different behavioral tendencies (e.g., playing video games), with different learning attitudes should differ in academic achievement. Therefore, we proposed the following hypotheses:

Hypothesis 2: Adolescents’ self-educational expectation mediates the relationship between video game playing and academic achievement.

Hypothesis 3: Adolescents’ learning attitude mediates the relationship between video game playing and academic achievement.

### 2.3 Parent-child relationship as a moderator

The family system, as a microsystem, plays an essential role in the growth of individuals. Aguilar-Yamuza et al. stated that the family serves as a key factor in preventing most internalizing problems during early childhood and adolescence [75]. Among the many factors of the family system, the parent-child relationship is an important one. It is the first interpersonal relationship that an individual perceives after birth, and the link between parent-child relationship and adolescent problem behavior is an important research topic in the field of developmental psychology [76, 77]. The parent-child relationship is usually defined as the relationship between a parent (primary caregivers, who can be a non-biological parent) and a child [78]. Moreover, the parent-child relationship is recognized as a foundational context for the formation of cyber literacy, which has a strong link to questionable internet use. Empirical research has supported the idea that adolescents who lack social relationships (e.g., parent-child relationships) are at a greater risk for problematic internet usage [79] and even internet addiction [80]. Hence, this study incorporated the parent-child relationship as a key factor.

Differences in the role of the parent-child relationship are also related to adolescents’ intrinsic motivation to learn. Previous studies revealed that a high level of parent-child interaction increases the expectations of both parents and children and that the higher shared family expectations, in turn, increase children’s achievement, while greater disparities in family expectations act as a disincentive [81]. That is, establishing a good parent-child relationship enables adolescents to feel love and respect from their parents and develop optimistic perceptions and expectations, which positively affects their physical and mental development and growth. Wang et al. also showed that family health is essential to a child’s mental health [82]. Nevertheless, a negative parent-child relationship often results in adolescents developing adverse perceptions and experiences towards their surroundings, such as depression [83]. In this view, we can assume that the parent-child relationship plays a moderating role in the effects of video games on adolescents’ academic achievement. Therefore, we proposed the following hypotheses:

Hypothesis 4: Parent-child relationship moderates the relationship between adolescent video game playing and academic achievement.
Hypothesis 5: Parent-child relationship moderates the mediating relationship of self-educational expectation between adolescent video game playing and academic achievement.
Hypothesis 6: Parent-child relationship moderates the mediating relationship of learning attitude between adolescent video game playing and academic achievement.

**Fig 1:**
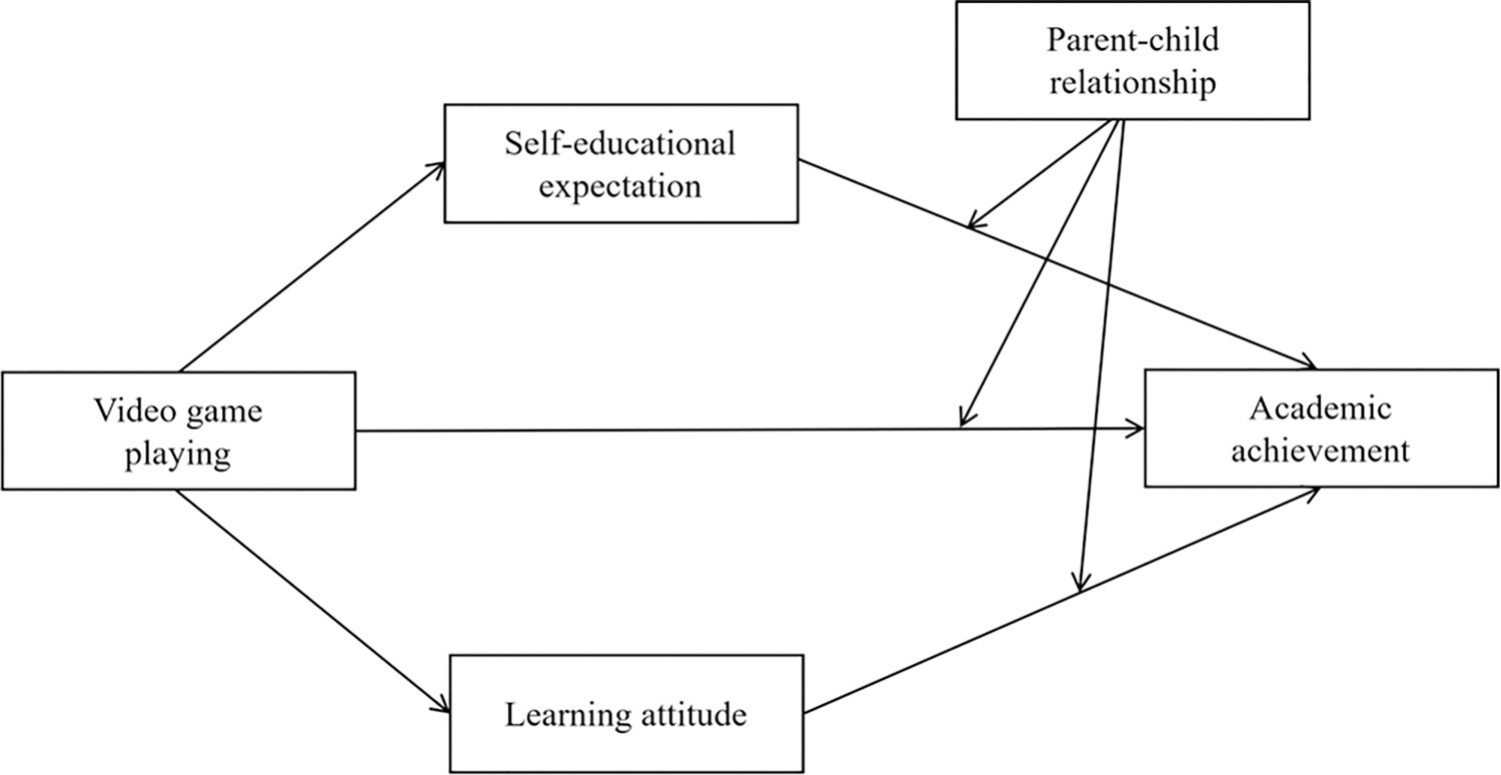
Proposed hypothesized model.

According to the above hypotheses, the current study proposes the following research model (See Fig 1).

## 3. Research methods

### 3.1 Participants

This study used follow-up data for 2014–2015 from the China Education Panel Survey (CEPS), conducted by the National Survey Research Center at Renmin University of China (NSRC). CEPS is the first nationwide and sustained large-scale tracking survey project that targets junior high school students in China. This project carried out the first round of baseline survey in 2013–2014, covering students in the first year of junior high school (Grade 7) and the third year of junior high school (Grade 9) at that time. This survey utilized a multi-stage probability-proportional-to-size sampling (PPS) method. With four stages of sampling, it selected 112 schools, 438 classes, and a total of 19,487 students across China for study, of which 10,279 were in Grade 7. Subsequently, students who were in Grade 7 in the 2013–2014 school year participated in a follow-up survey for the second round of data collection carried out in 2014–2015 (Grade 8 in this school year). The final sample of students successfully followed up was 9,449.

We then adjusted the sample according to the research goal of the present study. First of all, we obtained student demographic information, academic achievement for the 2013–2014 school year, and school information from the baseline survey results to supplement the data for the 2014–2015 school year. Next, considering that this study focuses on the influence of video game playing on adolescents’ academic achievement, we excluded students who never go to net bars or video arcades where they play online games (In China, this is a place designed for engaging in online and video gaming, drawing players from both adolescents and adults.), remaining a sample of 1,184. After data matching and removing missing samples for key variables, our study finally included 824 participants.

### 3.2 Measures

#### 3.2.1 Video game playing

The adolescents’ video game playing was measured by the question in the student-reported questionnaire of CEPS, namely “How often did you do the following things in the past year? Going to net bars or video arcades”. The original answers were categorized on a 5-point scale from 1 (never) to 5 (always). Since the present study did not include students who did not go to net bars or video arcades to play video games, we recorded the response to a 4-point scale where 1 = seldom, 2 = sometimes, 3 = often, and 4 = always.

#### 3.2.2 Self-educational expectation

The adolescents’ self-educational expectation was assessed using the question “What is the highest level of education you expect yourself to receive?” in the student-reported questionnaire of CEPS. To facilitate the subsequent analysis, we recorded the response as a 5-point scale where 1 = Junior high school, 2 = High school, 3 = Junior college, 4 = bachelor’s degree, and 5 = Master’s degree. A higher score means that the adolescent has a higher self-education expectation.

#### 3.2.3 Learning attitude

The learning attitude was measured by three questions of the CEPS that described the level of adolescents’ engagement and persistence in their studies. Adolescents were asked about the extent to which they agreed with the statements below: “I would try my best to go to school even if I was not feeling very well or I had other reasons to stay at home”, “I would try my best to finish even the homework I dislike”, and “I would try my best to finish my homework, even if it would take me quite a long time”. Each item was measured with a 4-point scale ranging from “1 = strongly disagree” to “4 = strongly agree”. Following Eisinga et al. [84], we calculated Spearman-Brown reliability estimates, which are more applicable to the two-to three-item scales. The Spearman-Brown reliability coefficient for learning attitude was 0.800, showing good reliability. The average score of these three items was calculated to reflect the adolescents’ learning attitude. A higher score indicates a higher level of learning attitude.

#### 3.2.4 Parent-child relationship

The parent-child relationship was assessed by two questions in the student-reported questionnaire of CEPS to reflect the relationship between adolescents and their father and mother, respectively. Adolescents were asked regarding their agreement with the following statements: “How is the general relationship between you and your father?” and “How is the general relationship between you and your mother?” Each answer was followed by a 3-point scale ranging from “1 = Not close” to “3 = Very close”. This scale had a relatively high reliability with a Spearman-Brown reliability coefficient = 0.666. An average score was calculated for these two items, with the higher score suggesting a better parent-child relationship.

#### 3.2.5 Academic achievement

The adolescents’ academic achievement was their performance during the 2014–2015 school year, which is measured by the 2015 midterm exam scores in Chinese, Math, and English provided in CEPS. The Spearman-Brown reliability coefficient for this scale was 0.837, showing satisfactory internal reliability. We added up the scores of these three items as a specific measure of adolescents’ academic achievement, with a higher score representing a high level of academic achievement.

#### 3.2.6 Control variables

Since adolescents’ academic achievement is influenced by factors at multiple levels, this study included individual, family, and school characteristics as control variables. Individual characteristics controlled the adolescent gender, only-child, score in Grade 7. Family characteristics controlled the family’s economic status, father’s educational level, and mother’s educational level. School characteristics controlled the region, ranking, type, and location of the school.

### 3.3 Statistical analyses

This study processed the collected data using SPSS 26.0. Altogether, data was analyzed in three steps. The first step was to perform descriptive statistics (including mean and standard deviation of the main variables) and correlation analysis. To examine the mediators of self-expectation and learning attitude and the moderator of parent-child relationship, the second step was to conduct parallel mediation analysis and moderated mediation analysis using the PROCESS macro v4.1 for SPSS. This not only tested whether the mediator variables could link the predictor variable (video game playing) to the outcome (academic achievement), but also assessed whether the direct and indirect effects varied by the moderator variable [85]. Finally, all mediated and moderated effects were tested using the Bootstrap method, with a setting of 5,000 bootstrap samples and 95% bias-corrected confidence intervals. If zero is not in the 95% CI, it means statistical significance [86]. In addition, simple slope plots were employed to draw the functions of different parent-child relationships [87].

## 4. Results

### 4.1 Preliminary analyses

Table 1 provides the descriptive statistics and Pearson correlation matrix for the variables of video game playing, self-educational expectation, learning attitude, parent-child relationship, and academic achievement. The results showed that the correlation coefficients between all the key variables were significant, except between parent-child relationship and academic achievement. Specifically, adolescent video game playing was negatively correlated with self-educational expectation (r = -0.119, p < 0.01), learning attitude (r = -0.205, p < 0.001), parent-child relationship (r = -0.116, p < 0.01), and academic achievement (r = -0.152, p < 0.001). Self-educational expectation was positively associated with learning attitude (r = 0.275, p < 0.001), parent-child relationship (r = 0.078, p < 0.05), and academic achievement (r = 0.484, p < 0.001). In addition, learning attitude was positively correlated with both parent-child relationship (r = 0.157, p < 0.001) and academic achievement (r = 0.254, p < 0.001). However, parent-child relationship was not significantly correlated with academic achievement (r = 0.033, p > 0.05).

**Table 1:** Descriptive statistics and correlation matrix of key variables.

Variables	M	SD	Video game playing	Self-educational expectation	Learning attitude	Parent-child relationship	Academic achievement
Video game playing	1.561	0.881	1				
Self-educational expectation	3.075	1.228	-0.119**	1			
Learning attitude	2.767	0.768	-0.205***	0.275***	1		
Parent-child relationship	2.511	0.495	-0.116**	0.078*	0.157***	1	
Academic achievement	187.992	71.465	-0.152***	0.484***	0.254***	0.033	1

Note

* p-value < 0.05

** p-value < 0.01

*** p-value < 0.001.

### 4.2 Test for the parallel mediation effect

Table 2 presents the results of the mediating and moderating effects. First, Model 1 tested the total effect of adolescents’ video game playing on academic achievement. Second, Model 2 and Model 3 examined the effects of adolescents’ video game playing on self-educational expectation and learning attitude respectively. Third, Model 4 in the PROCESS macro was used to develop Model 4 (in Table 2) to examine whether there was a direct effect of the two mediating variables (self-educational expectation and learning attitude) on academic achievement as well as to confirm whether there was a direct effect of video game playing on academic achievement. At this point, the mediating effect test was completed. Finally, Model 15 in PROCESS macro was used to develop Model 5 to determine whether the moderating effect of the parent-child relationship was significant.

**Table 2:** Conditional process analyses.

Variables	Model 1 AA		Model 2 SEE		Model 3 LA		Model 4 AA		Model 5 AA	
	β	t	β	t	β	t	β	t	β	t
Grade 7 score	0.645	25.055***	0.363	11.470***	0.179	5.221***	0.553	20.794***	0.547	20.466***
Gender	0.004	0.148	0.054	1.893	0.037	1.081	-0.011	-0.435	-0.012	-0.474
Only-child	0.100	3.684***	0.036	1.022	0.046	1.261	0.089	3.426***	0.091	3.492**
Economic status	0.009	0.353	-0.073	-2.229*	-0.052	-1.484	0.029	1.146	0.029	1.128
Father’s education	0.046	1.404	0.159	3.951***	0.071	1.624	0.006	0.192	0.009	0.291
Mother’s education	0.032	0.940	0.015	0.724	-0.069	-1.521	0.034	1.044	0.035	1.090
School region	-0.179	-6.823***	-0.068	-2.105*	-0.056	-1.592	-0.160	-6.376***	-0.165	-6.578***
School ranking	0.133	5.080***	0.174	5.389***	-0.017	0.477	0.097	3.792***	0.090	3.508**
School type	-0.062	-2.333*	-0.034	-1.036	0.066	1.853	-0.060	-2.342*	-0.052	-2.011*
School Location	0.029	1.055	0.065	1.902	-0.005	-0.122	0.016	0.585	0.012	0.457
VGP	-0.096	-3.609***	-0.094	-2.992**	-0.201	-4.000***	-0.061	-2.440*	-0.065	-2.604**
SEE							0.217	7.799***	0.219	7.833***
LA							0.075	2.914**	0.082	3.171**
PCR									-0.024	-0.964
VGP*PCR									-0.050	-2.051*
SEE*PCR									0.055	2.118*
LA*PCR									-0.026	-0.999
R^2^	0.489		0.227		0.093		0.537		0.542	
F	64.666***		19.825***		6.896***		66.881***		52.919***	

Note

* p-value < 0.05

** p-value < 0.01

*** p-value < 0.001.

**Abbreviations:** AA = Academic achievement, VGP = Video game playing, SEE = Self-educational expectation, LA = Learning attitude, PCR = Parent-child relationship.

In Hypothesis 1, we assumed that video game playing would negatively affect the academic achievement of adolescents. According to Model 1 (Table 2), academic achievement was significantly predicted by video game playing (β = -0.096, p < 0.001). Meanwhile, the results of Model 4 with mediating variables reconfirmed this negative relationship. Therefore, Hypothesis 1 was supported.

Hypothesis 2 predicted that self-educational expectation would mediate the effect of video game playing on academic achievement. According to Models 2 and 4 (Table 2), video game playing negatively related to self-educational expectation (β = -0.094, p < 0.01), and self-educational expectation positively predicted academic achievement (β = 0.217, p < 0.001). The results of bootstrap in Table 3 indicate that through self-educational expectation, the indirect effect of video game playing on academic achievement was significant and negative (β = -0.020, p < 0.01, 95%CI = [-0.037, -0.006]). Therefore, Hypothesis 2 was supported.

**Table 3:** Testing the pathways of the parallel mediation model.

Path	Effect value	Relative effect quantity	Bootstrap (95%CI)
Video game playing → Self-educational expectation → Academic achievement	-0.094×0.217 = -0.020	20.83%	[-0.037, -0.006]
Video game playing → Learning attitude → Academic achievement	-0.201×0.075 = -0.015	15.63%	[-0.027, -0.005]
Total mediation effect	-0.035	36.46%	[-0.056, -0.017]
Direct effect	-0.061	63.54%	-

Hypothesis 3 predicted that learning attitude would mediate the effect of video game playing on academic achievement. As can be seen in Models 3 and 4 (Table 2), video game playing negatively correlated with learning attitude (β = -0.201, p < 0.001), and learning attitude positively predicted academic achievement (β = 0.075, p<0.01). Combined with the bootstrap results in Table 3, it can be seen that through learning attitude, the indirect effect of video game playing on academic achievement was significant and negative (β = -0.015, p < 0.01, 95%CI = [-0.027, -0.005]). Therefore, Hypothesis 3 was supported. In addition, the total mediation effect (-0.035) accounted for 36.46% of the total effect (-0.096).

### 4.3 Test for the moderated mediation effect

**Fig 2:**
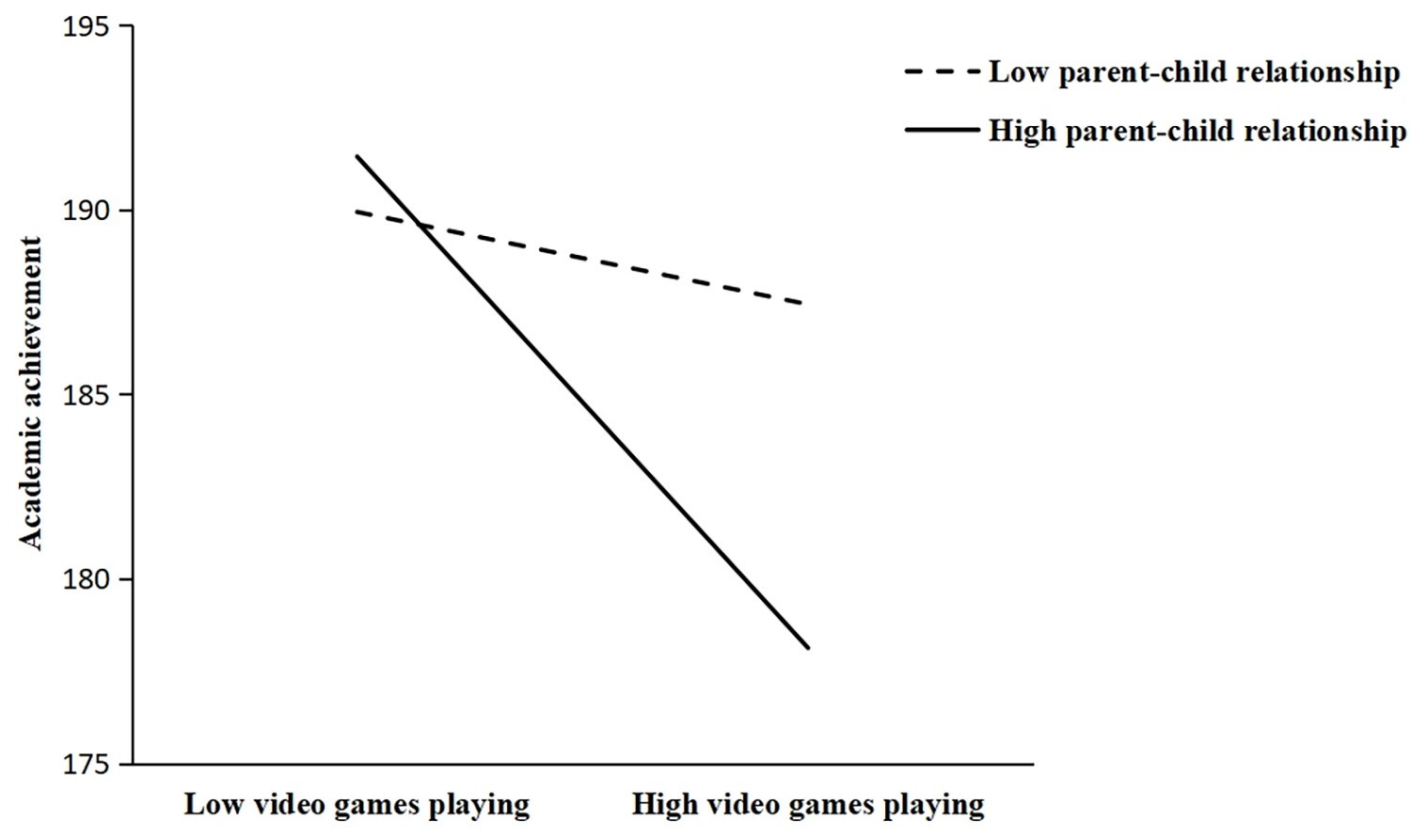
Parent-child relationship as a moderator between video game playing and academic achievement.

Hypothesis 4, Hypothesis 5, and Hypothesis 6 predicted that parent-child relationship would moderate the direct and indirect effect of video game playing on adolescent academic achievement. In Hypothesis 4, we assumed that the parent-child relationship would moderate the direct association between video game playing and academic achievement. As is demonstrated in Model 5 (Table 2), the interaction term between video game playing and parent-child relationship had a significant impact on academic achievement (β = -0.050, p < 0.05). It means that the parent-child relationship enhanced the negative relationship between video game playing and academic achievement. Therefore, Hypothesis 4 was supported. To further describe the nature of the supported moderation, we plotted predicted academic achievement against video game playing separately for different levels of parent-child relationship. The results are shown in Fig 2. It is evident that among adolescents with high parent-child relationship, there was a somewhat strong correlation between video game playing and academic achievement (b = -9.227, p < 0.01), but not for adolescents with low parent-child relationship (b = -1.735, p = 0.502 > 0.05).

**Fig 3:**
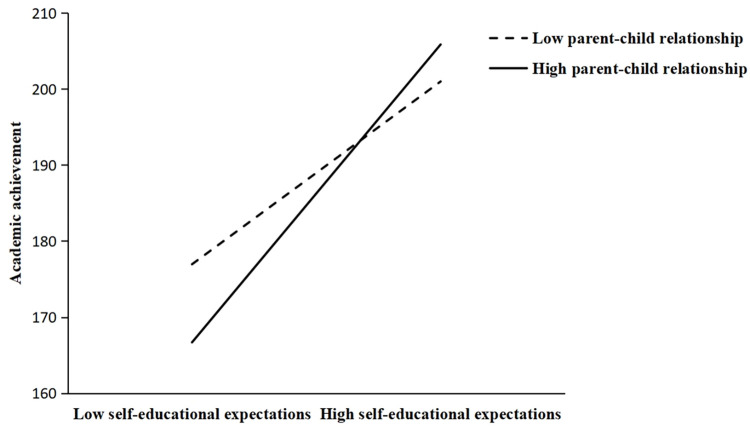
Parent-child relationship as a moderator between self-educational expectation and academic achievement.

In Hypothesis 5, the current study expected that the indirect relation between video game playing and academic achievement via self-educational expectation was moderated by the parent-child relationship. The results of Model 5 (Table 2) show that there was a significant interaction between parent-child relationship and self-educational expectation in predicting academic achievement (β = 0.055, p < 0.05), which means that parent-child relationship played a positive moderating role in the impact of self-educational expectation on academic achievement. The bootstrap results further indicated that there was a stronger indirect relation between video game playing and academic achievement for adolescents with high-quality parent-child relationship (b = -2.091, 95%CI = [-3.802, -0.570]), and this indirect relation became weaker for those with low-level parent-child relationship (b = -1.283, 95%CI = [-2.506, -0.312]). Therefore, Hypothesis 5 was supported. For descriptive purposes, we graphed a simple slope plot, see Fig 3.

**Fig 4:**
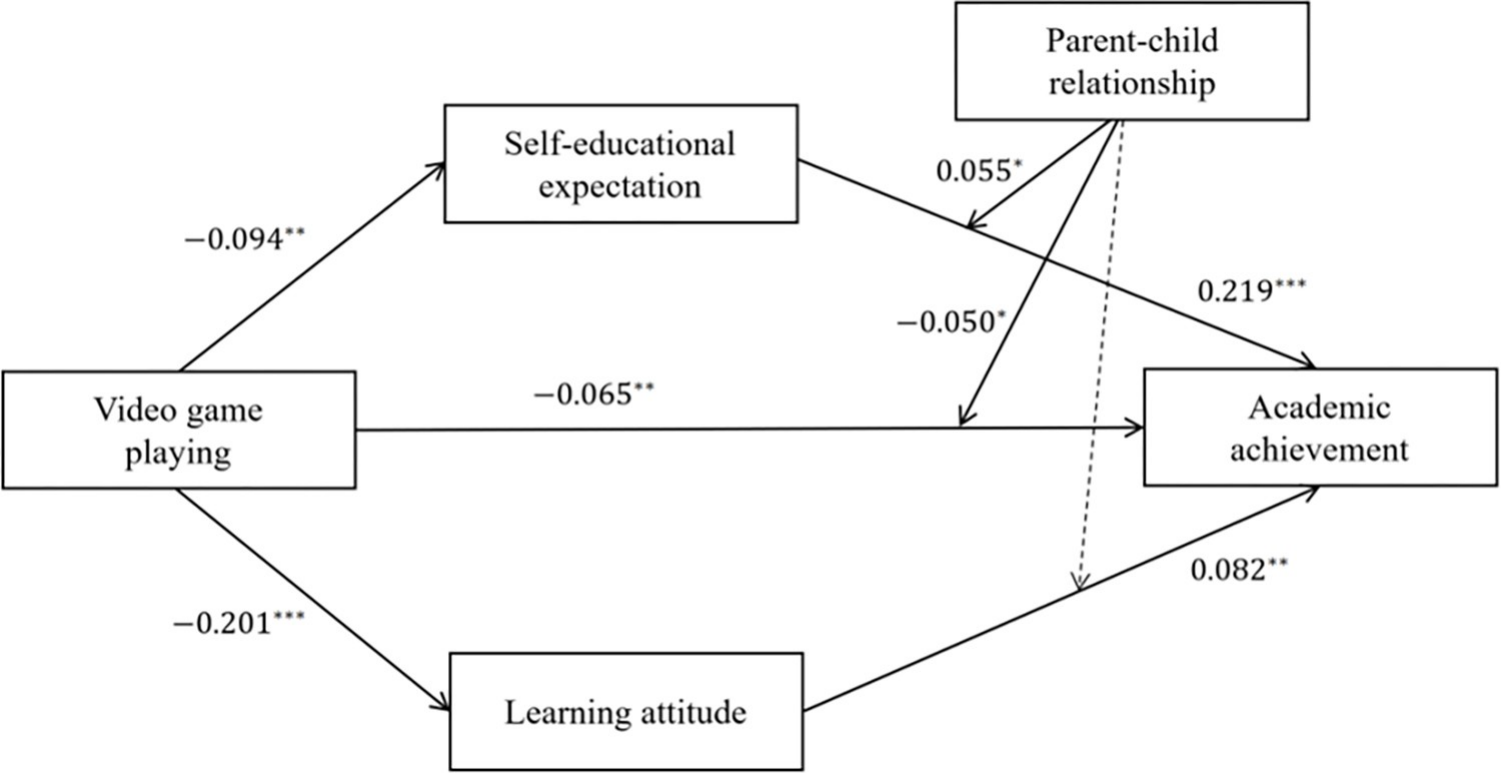
Moderated-mediation model. Note: *p-value<0.05, **p-value< 0.01, *** p-value<0.001.

In Hypothesis 6, we expected that the indirect relation between video game playing and academic achievement via learning attitude was moderated by parent-child relationship. As is depicted in Model 5 (Table 2), the interaction term of learning attitude and parent-child relationship had a non-significant impact on academic achievement (β = -0.026, p = 0.318 > 0.05). Therefore, Hypothesis 6 was not supported. The overall model accounted for 54.20% of the variance in adolescent academic achievement. Furthermore, the moderated mediation model among the main variables is shown in Fig 4.

## 5. Discussion

Based on the social cognitive theory, this study explored the direct and indirect impacts of video game playing on academic achievement using a representative dataset from CEPS. The findings showed that video game playing is a significant but modest predictor of academic achievement among Chinese adolescents. To evaluate the poorly defined mechanisms by which video game playing predicts academic achievement, two mediators of self-educational expectation and learning attitude were considered and the moderating role of parent-child relationship was also explored. By showing parallel mediation impacts of learning attitude and self-educational expectation, together with the moderating influence of parent-child relationship, our findings generally supported research hypotheses.

### 5.1 Relationship between video game playing and academic achievement

Our study first revealed that video game playing is a factor worth considering when predicting academic achievement among Chinese adolescents, although its effect size is not very strong. Specifically, the higher the degree of Chinese adolescents’ video game playing, the less likely they are to achieve good academic achievement. This is consistent with the previous empirical studies in different countries [25–27, 44]. The impact of video games on academic achievement is not absolute, and moderate recreation has certain positive effects on young people. However, if adolescents frequently visit internet cafes to play video games, they could end up spending more time there than is appropriate, which would interfere with their ability to balance study and relaxation. Due to the heavy academic load of adolescents, if they devote a lot of time to video games [27], or even suffer from game addiction [22, 28], it could lead to lower academic achievement. Moreover, with the rapid development of internet technology and smart devices, it has become more and more convenient for adolescents to play video games, and it is even common for families to have good access to the internet. Therefore, the negative impact of excessive video games on adolescents’ academic achievement deserves more attention in various countries, including China.

### 5.2 Parallel mediating role of self-educational expectation and learning attitude

Our results revealed that the parallel mediation of self-educational expectation and learning attitude in the association between video game playing and academic achievement was statistically significant. Numerous studies have been conducted to explore the impact of the internet and video games on students’ academic achievement, but the mechanism underlying this effect has received less attention. As the social cognitive theory mentioned above emphasizes that external factors influence individual behavior by acting on self-processes [63], the findings of this study respond to this view and supplement a new perspective. Both self-educational expectation and learning attitude are a reflection of an individual self-perception process. Adolescents who are immersed in video games may experience a gradual decline in their enthusiasm for learning, which can undermine their academic expectations and motivation, ultimately negatively impacting their academic achievement. This is in line with Zhang et al.’s study that internet addiction reduces student engagement in learning, increases dissatisfaction with learning activities, and thus lowers academic achievement [35]. Specifically, several factors related to video games may contribute to the sustained damaging consequences on academic achievement. For instance, adolescents who are addicted to video games gradually lose control over their internet use, thereby reducing the time and energy they devote to their studies [27, 88]. Teenagers with high levels of internet addiction can easily experience negative emotions, such as moodiness, anger, anxiety, and sadness, which make them feel bored and withdrawn from the world outside the internet [89]. Moreover, the immersive environment of online games may sometimes lead adolescents to passively engaging with the virtual world, potentially impacting their autonomy and decision-making skills [19, 40]. All of these ultimately make it difficult for adolescents to focus on study, which in turn may cause a decline in their academic achievement. To address these challenges, parents work with teachers to help adolescents find a balance between video games and academics, and encourage them to actively participate in academic and real-life activities, which will be conducive to fostering their self-awareness and promoting their all-round development [90].

Furthermore, the results showed that the mediating effect of self-educational expectation accounted for a greater proportion than learning attitude. This implies that adolescents’ video game playing in particular affects academic achievement by influencing their educational expectations. It can be explained by a psychological difference. Lim and Reeves pointed out that online game worlds typically contain elements such as quests, rankings, incentives, and upgrades [91]. Each action is accompanied by immediate feedback like rewards or punishments, which results in a straightforward but effective psychological control mechanism that makes it possible for the players to enjoy themselves. In contrast, adolescents may put in months of intense learning before they can gain a sense of accomplishment on an exam. The instant gratification provided by such games contrasts sharply with the relatively long payback period of the learning process. As this psychological discrepancy grows, adolescents develop a sense of aversion to learning and lower expectations for education. In addition, adolescents’ self-educational expectation, as the main motivation for self-worth realization and thinking ability innovation, is a more permanent and long-term goal setting, and its influence on academic development is more persistent. However, learning attitude, on the other hand, is more inclined to current and short-term tasks, and it is more susceptible to changes in the external environment and tasks, thus its impact on academic achievement is likely to be relatively short-lived. Therefore, we introduced two mediating variables from individual perspectives, especially self-educational expectation, to make the findings more thought-provoking.

### 5.3 Moderating role of parent-child relationship

The present study indicated that parent-child relationship plays a slightly moderating role in the influence of video games on adolescents’ academic achievement, whether directly or indirectly. In particular, the parent-child relationship moderated the direct association between video game playing and academic achievement, as well as the indirect association between video game playing and academic achievement via the mediator of self-educational expectation. However, the relationship of video games affecting academic achievement through learning attitude did not differ across parent-child relationship levels. The results highlight that the relationships and interactions between parents and children are important emotional regulators in children’s development [92, 93]. Overall, compared with a low parent-child relationship, the video game playing of adolescents with a high parent-child relationship has a greater impact on academic achievement.

First of all, our findings revealed that the association between video game playing and academic achievement was significant for adolescents with a high level of parent-child relationship, while it was not significant among the low-level parent-child relationship group. This study yielded a different finding that adolescents with high levels of parent-child relationships performed the worst academically when playing video games frequently. A possible explanation is that positive parent-child relationships may lead parents to adopting a more relaxed parenting style, viewing gaming as a shared activity and giving adolescents greater freedom to play video games, rather than strictly limiting their gaming time [94]. Previous literature has confirmed that parents’ positive attitudes toward media use is positively correlated with adolescents’ internet use and gaming playing [78, 95]. In a close parent-child relationship, the relaxed regulation of gaming by parents may provide adolescents with greater freedom, potentially resulting in excessive gaming that negatively affects their academic achievement. In contrast, parental interference with adolescent autonomy and excessive control is often linked to poor parent-child relationships (e.g., parent-child conflict) [96]. In accord with this, parents may impose stricter rules on video game use to manage their children’s behavior, which could weaken the relationship between video games and adolescents’ academic achievement. This inspires us to include parenting styles and parental supervision in future studies when exploring the impact of video games on academic achievement.

Moreover, the results further confirmed that parent-child relationship modestly moderated the association between video game playing and academic achievement through self-educational expectation. For adolescents with a better parent-child relationship, this indirect link was greater than for the low parent-child relationship group. That is, the positive effect of adolescents’ self-educational expectation on academic achievement is strengthened by the improvement of the parent-child relationship. Adolescence is a crucial period for a person to create identity and develop values [29]. Aligning with the attachment theory, a positive parent-child relationship can fulfill the needs of adolescents and thus facilitate the formation of positive representations of themselves and the environment [97]. In families characterized by low-quality parent-child relationships, children may suffer more negative emotions (e.g., depression) and exhibit adverse social adaptation. These factors are harmful to the development of children’s educational expectations. Meanwhile, parental educational aspirations have been identified as a key factor influencing children’s educational aspirations [98]. With poorer parent-child relationships, parents are likely to exhibit low expectations of the value and importance of education, thereby lowering children’s expectations. On the contrary, good parent-child relationships can minimize the detrimental effects of video games on academic achievement by giving teenagers a safe space where their basic psychological needs can be satisfied. This helps them build positive goals and expectations. Therefore, the role of the parent-child relationship should be taken into account. Nevertheless, the current study indicated that no support was shown for the moderation of parent-child relationship in the association between video game playing and academic achievement through learning attitude. One explanation may be that the physiological and psychological changes that occur during adolescence, together with the intricate social environment, continuously enhance adolescents’ autonomy [29]. Hence, the influence of families in shaping learning attitudes becomes more complex. Previous research suggested that family, school, peers, and even neighbors are all significant factors in influencing adolescents’ internet use [99]. It implies that adolescents’ attitudes toward academics are also susceptible to such multiple external factors, and thus the parent-child relationship may show relatively limited effects in enhancing children’s learning attitude. This inspires us to think more about the important role of parent-child relationship in the deep cognitive level of adolescent self-educational expectation in future research on adolescent video games and even internet use.

## 6. Limitations and implications

To summarize, the current study has the following limitations. First, this study was a cross-sectional design using the data from the survey conducted by CEPS in 2014–2015, thereby constraining our ability to make causal inferences about the findings. A longitudinal study should be adopted in future research to delve deeper into causal relationships and provide a more comprehensive assessment of the impact of video games on adolescents’ academic achievement, as well as to elucidate the underlying mechanisms. Second, the data of this study is secondhand data, some of the variables were measured through relatively limited items. Thus, future measures of video game playing could be more quantified in a measurable way like how many times a week or how many hours a week to avoid subjective bias. Future research could improve measurement accuracy by employing more comprehensive scales, such as the parent-child relationship inventory (PCRI) [100]. Moreover, with the rapid development of internet technology, video games are no longer limited to traditional internet cafes and are now easily accessible on various mobile devices that can be used conveniently at home. Therefore, future studies should use the most up-to-date data to measure video game playing among adolescents, and may even go further to differentiate the specific effects of video games and other internet use, such as smartphones [101, 102], on academic achievement. Third, CEPS used a self-report questionnaire to measure students’ video game playing, learning attitude, self-educational expectations, and parent-child relationship, which may cause response biases. Thus, future studies should use more objective data collection methods to enhance the validity and reliability of the findings. Additionally, it is important to gather information from adolescents, parents, and teachers for a more thorough evaluation of these variables. Fourth, the sample of this study was limited to junior high school students in the teenage group, but adolescents from different age and grade groups exhibit distinct developmental stages and are exposed to extremely different peer groups and educational contexts. Therefore, future research should include adolescents from different sub-groups to further test the reliability and applicability of the findings of this study. Finally, although this study explored the mechanisms of video game influence on adolescents’ academic achievement from the perspective of students’ self-perception and parent-child relationship, there are still some unmeasured potential confounding variables that may affect the interpretation of the results. For example, parenting style and parent-child communication quality at the family level [103–105], peer relationships and school climate at the social support level [106–108], and individual characteristics like gender difference, self-control [109–111], have been proven to be significantly associated with children’s video game use and academic achievement. Therefore, future studies need to further explore and identify additional potential factors, including family environment, social support, individual psychological characteristics. By integrating these factors to reveal more comprehensively the mechanisms affecting adolescents’ academic achievement, the understanding in this area can be enriched and deepened.

Despite these limitations, the findings of the present study provide several theoretical and practical implications for future studies to explore the relationship between adolescent video game playing (even internet use) and academic success. Theoretically, the moderated mediation model proposed by this study, based on social cognitive theory, provides a possible theoretical framework for future research to explore the direct and indirect relationships between video game playing and adolescents’ academic achievement. Although previous studies have also explored individual and family factors in the relationship between video games and academic achievement in the West [4, 37, 44, 112], the current study extended the literature by indicating the mediating role of individual factors (e.g., self-educational expectation and learning attitude) and the moderating role of family factors (e.g., parent-child relationship) in one model based on research in China. In Western countries, child-rearing may place greater emphasis on independence and personal choice, while in East Asian cultures, family expectations and social pressures can have a more significant impact on children’s educational pursuits [113, 114]. From this perspective, this study enriches current knowledge by providing robust evidence from a national sample of Chinese adolescents. Moreover, it suggests a theoretical perspective for exploration of the interaction between family, individual, and social factors, as well as valuable insights for future comparative research in different contexts.

In terms of practical implications, a major implication of this study is that key stakeholders (e.g., parents, teachers, and policymakers) should provide balanced guidance on video game use, while fostering positive family relationships to support adolescents’ educational expectation and learning attitude, ultimately mitigating the negative impact of excessive gaming on academic achievement. Specifically, our findings revealed that adolescent self-educational expectation and learning attitude were mediating factors of video game playing to reduce academic achievement. Adolescence is a crucial developmental stage during which individuals begin to establish their values and worldviews. This period is characterized by heightened vulnerability due to potential disconnections among the developing brain, behavioral patterns, and cognitive systems [115]. As a result, adolescents become particularly sensitive to external influences, which can significantly impact their development and overall well-being. Thus, the effects of excessive video games on adolescents’ academic achievement and even personal development are not only immediate, but also subconsciously eliminate their correct perceptions of academic pursuits. It reminds parents and teachers to attach importance to the potential long-term effects of video games on the cognition of adolescents. Educators could offer courses on time management and self-regulation training to help students cultivate a positive learning attitude and self-educational expectations, effectively guiding them in balancing their study and recreation. In addition, this study found that the parent-child relationship moderated the influence of adolescents’ video game playing on academic achievement to some extent, including both the direct effect and the indirect effect through self-educational expectation. Previous studies have demonstrated the effectiveness of a good parent-child relationship in preventing internet addiction [116], but parents may face the challenge of encouraging their children to utilize online resources fully while protecting them from harmful content [117]. Our findings further suggest that educators and parents should focus on fostering a supportive parent-child relationship, combined with reasonable supervision. A positive self-educational expectation of adolescents can be fostered by providing family guidance and assisting children to strike a good balance between recreational activities and academics.

## 7. Conclusions

Considering the significance of individual traits, this study aimed to clarify the possible mechanisms by which video game playing influences adolescents’ academic achievement from the perspective of social cognitive theory. By analyzing data from a comprehensive survey in China, the study took an important step in exploring the roles of self-educational expectation, learning attitude, and parent-child relationship and provided valuable insights regarding preventing the adverse effects of excessive gaming on adolescents’ academics. The results revealed that self-educational expectation and learning attitude play a parallel mediating role in the association between video game playing and academic achievement. Moreover, parent-child relationship moderated the direct effect of video game playing on academic achievement and the indirect effect of video game playing on academic achievement via self-educational expectation. While the moderating role of parent-child relationship in the association between learning attitude and academic achievement was not significant. This study highlights the importance of fostering accurate academic perceptions and positive attitudes in adolescents to enhance their academic achievement. Further, guidance provided by various stakeholders to strengthen parent-child relationships is also needed to benefit the academics of adolescents.
